# Structural dynamics of tight junctions modulate the properties of the epithelial barrier

**DOI:** 10.1371/journal.pone.0214876

**Published:** 2019-04-09

**Authors:** Aapo Tervonen, Teemu O. Ihalainen, Soile Nymark, Jari Hyttinen

**Affiliations:** Faculty of Medicine and Health Technology and BioMediTech Institute, Tampere University, Tampere, Finland; Emory University School of Medicine, UNITED STATES

## Abstract

Tight junctions are dynamic structures that are crucial in establishing the diffusion and electrical barrier of epithelial monolayers. Dysfunctions in the tight junctions can impede this barrier function and lead to many pathological conditions. Unfortunately, detailed understanding of the non-specific permeation pathway through the tight junctions, the so-called leak pathway, is lacking. We created computational models of the leak pathway to describe the two main barrier measures, molecular permeability and transepithelial electric resistance while using common structural dynamics. Our results showed that the proposed alternatives for the leak pathway, the bicellular strand opening dynamics and the tricellular pores, contribute together with distinct degrees, depending on the epithelium. The models can also capture changes in the tight junction barrier caused by changes in tight junction protein composition. In addition, we observed that the molecular permeability was markedly more sensitive to changes in the tight junction structure and strand dynamics compared with transepithelial electric resistance. The results highlight that our model creates a good methodological framework to integrate knowledge on the tight junction structure as well as to provide insights and tools to advance tight junction research.

## Introduction

Epithelial cell monolayers cover body surfaces and line different organs. These tissues separate the underlying organs from their surroundings by creating tight barriers, and cell-cell junctions play a crucial role in this process. The most significant components for the barrier function are the tight junctions (TJs). These dynamic structures bring the membranes of adjacent cells into close contact, and thus seal the paracellular space between them. Due to their important role in the epithelial function, it is not surprising that there are several diseases, such as inflammatory bowel disease and celiac disease, which are linked to dysfunctions in TJ proteins or in the TJ complexes themselves [1, 2]. In these pathological conditions, the epithelium usually becomes leaky [3], and thus rendering it unfit for its task. In the present work, we investigate the dynamic properties of the epithelial barrier by developing a computational model of TJ structure.

**Fig 1:**
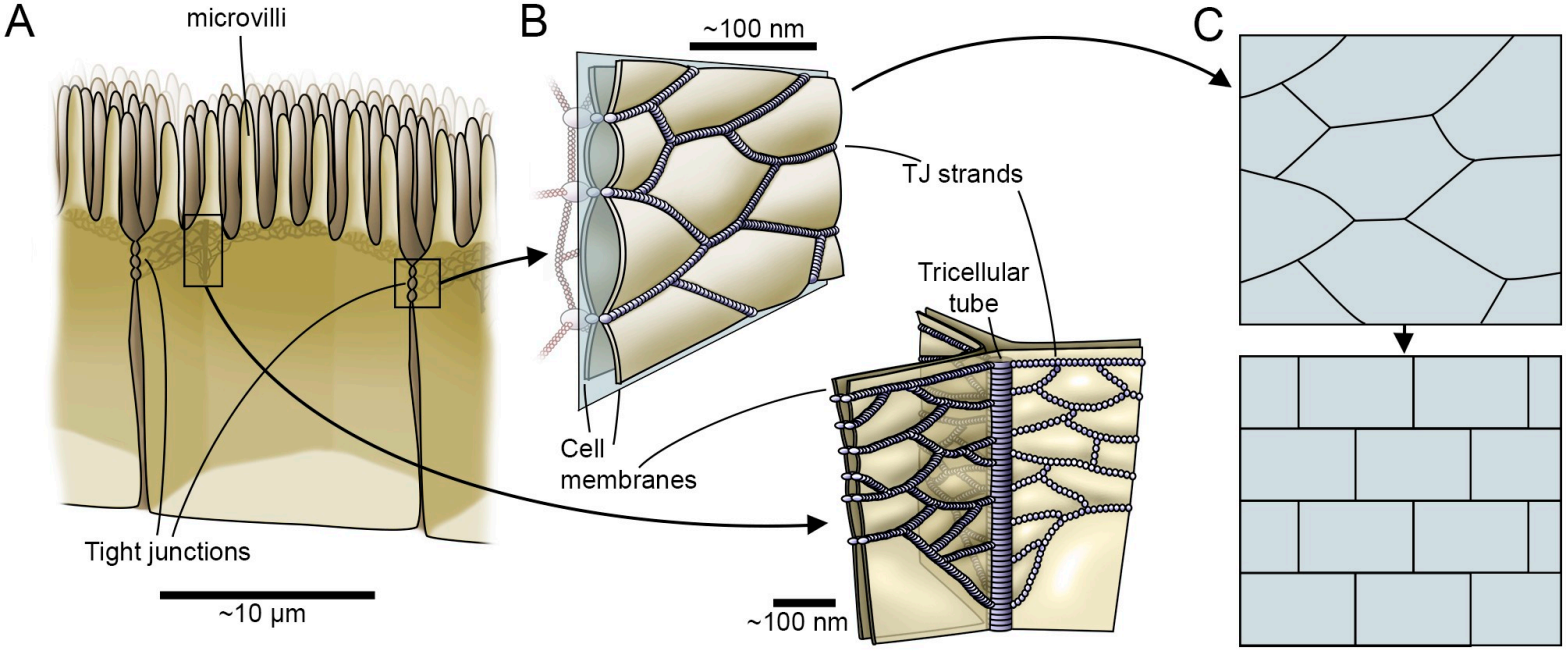
Tight junction structure and model simplification. (A) A schematic figure showing how the tight junctions (TJs) encircle the apical end of the paracellular space between the epithelial cells, and thus form the barrier. (B) Close up of the bTJ strands (top) that form a network dividing the space between the cells into compartments and the tTJ central tube at the intersection of three cells (bottom). (C) A 2D depiction of the TJ strands and the compartment ultrastructure, based on the cutting plane shown in b. This structure is further simplified to form the 2D TJ strand network used in the model.

In epithelia, TJs encircle the cells near the apical side, as shown in Fig 1A, and they are categorized into bicellular and tricellular junctions [4]. Bicellular TJs (bTJs) appear as a network of anastamosing strands on the cell membranes between two cells [5, 6] and tricellular TJs (tTJs) form at the intersections of three cells [7] (Fig 1B). Generally, the bTJ strands are considered to be composed of different proteins; however, it has been proposed that lipids might have an important role in TJ formation [8–12]. According to the protein model, the strands comprise transmembrane proteins that bind to the respective proteins in the neighboring cells [13, 14]. The main transmembrane proteins in the strands are claudins, of which there are several types, and occludin [13, 15]. These proteins are connected to the actomyosin cytoskeleton via scaffolding proteins, such as ZO-1 [13]. Near the tTJs, the bTJ strand network extends vertically and converges near a 10-nm-diameter central tube [4, 7, 16]. It is not completely understood how these proteins form the strands or how the lipids might fit into this ensemble [11–13]. However, progress has been made as structural bTJ strand models were recently proposed [14, 17] based on the claudin crystal structure [18, 19].

TJ tightness varies between epithelial tissues and tasks [20]. These differences arise, for example, from the number of strands [21] and the bTJs’ protein composition, which has a large impact on the ion permeability of the strands [22]. Additionally, TJ barrier is not a static structure, since it is regulated by changes in the TJ protein composition and through the actomyosin cytoskeleton [23, 24].

The properties of the epithelial barrier are usually characterized by how ions and small molecules permeate through the epithelium [25]. Transepithelial electrical resistance (TER) is the most straightforward measurement of the ion permeability since it simply measures the instantaneous electrical resistance of the tissue [26]. In low-TER epithelia, the TJs define the total epithelial resistance due to the considerably higher resistance of the transcellular pathway formed by the cell bodies [27–30]. However, the transcellular component becomes more significant as the TER increases. The permeability of noncharged molecules has been approached by studying how small tracer molecules, such as polyethylene glycols (PEGs) [31–36], dextrans [16, 37, 38], and mannitol [33, 39], traverse the epithelial barrier. Especially different sizes of PEGs have been an invaluable tool when studying how molecular size (<1 kDa) affects the TJ permeation [31–36], since they have been shown to permeate paracellularly [40, 41].

Molecules and ions are hypothesized to permeate through the TJs via two routes: pore and leak pathways [31, 33, 42, 43]. Although these pathways were defined based on the permeability of noncharged molecules [31, 33], they can be extended to the context of the TER. The pore pathway is a high-throughput pathway for molecules with a radius smaller than 0.4 nm through pores formed by some claudins [31–33]. The ion permeability of these pores depends on the ion-specificity of the claudins forming them [44]. The leak pathway is a nonspecific, low-throughput pathway with an assumed size limit of over 6 nm [20, 31, 33, 45]. The origin of this pathway is debated, the two main candidates being 1) the pores in the tTJ central tubes and 2) transient bTJ strand breaks [16, 20, 22, 33, 45–48]. The tTJ pores have been shown to enable macromolecule diffusion: Krug et al. [16] showed that 3-kDa dextran passes through the epithelium via the tTJ pores and suggested that they could form the leak pathway. They further showed that changes in the expression of tricellulin, a TJ protein found especially in the tTJs, affected the permeability of macromolecules, but only minor changes in the permeability of small (<1 kDa) molecules or in TER were seen [16].

The bTJ strands have been shown to remodel constantly at the protein-level [43, 49] as well as at the strand-level in transfected fibroblasts by constant strand breaking and sealing events [46, 50]. The bTJ strand breaks have also been observed in freeze fracture electron microscope images [16, 51–53]. Furthermore, Van Itallie and coworkers recently showed that the connection between ZO-1 and claudins stabilizes the movement of the strands while, interestingly, not affecting the break frequency [50]. In addition, ZO-1 knockdown and double ZO-1/2 knockdown have been demonstrated to increase the leak pathway permeability while only slightly affecting the TER [34, 54]. Based on these findings, it has been suggested that structural dynamics in the strands could enable a step-by-step passage for molecules too large to pass through claudin pores. However, the slow dynamics in the strand structure would not be visible in the almost-instantaneous TER measurement [22, 33, 34, 46, 48]. Moreover, Liang & Weber [20] suggested that these two pathways are not mutually exclusive.

In recent years, experimental research on the TJ structure and function has been abundant and many important advances have been made [14, 18, 55–58]. However, the small size of the TJs and the molecules passing through them, together with the fast time scales of the events, make TJs and especially their structural dynamics challenging to study experimentally. Computational models complement experimental work and provide an excellent tool to investigate the characteristics of the TJ barrier in more detail. Most of the previous computational models of the TJ barrier [47, 59–64] describe a simplified and static TJ structure and do not include any level of structural dynamics. Weber and coworkers have constructed models describing the transient opening behavior of the claudin pores without the strand-level dynamics [65, 66]. To the best of our knowledge, the only computational model to include the strand dynamics is a percolation analysis model of the TJ strands as a random resistor network by Washiyama et al. [67]. However, a model that describes the structural TJ dynamics in relation to both the molecular permeability and the TER seems to be lacking.

In this work, we developed computational models for both the molecular permeability and the TER with common structural dynamics to investigate the origin of the leak pathway. We fitted the models to experimental data from two strains of MDCK monolayers and Caco-2 monolayer to parametrize the TJ structure. This, in addition to studying the leak pathway, enabled us to investigate how the different properties of this dynamic system affect the molecular permeability and the TER. With our models, we aim to fill the gap in knowledge between the structural and the functional properties of the TJs.

## Model description

### Modeling framework

**Fig 2:**
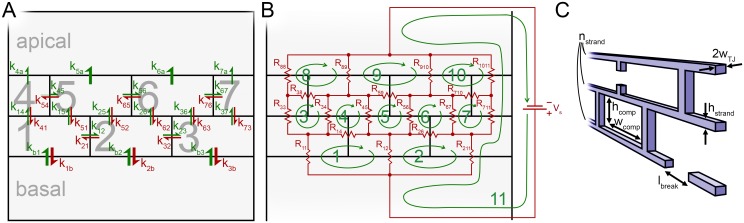
Schematic description of the molecular permeability and TER models. Schematic descriptions of the molecular permeability and the TER models as well as the geometrical parameters. An example of the geometrical idea of the models with three strands and the width of three compartments. (A) The molecular permeability model comprises bTJ compartments (numbered 1–7) lined by the TJ strands and the basal and apical compartments below and above the TJs, respectively. Rate constants *k*_*ij*_ describe the rate of permeation from compartment *i* to *j*. We assume low concentration in the apical compartment, and thus omit the backflow into the small compartments. To describe the strand breaks, the rate constant values are varied based on given probabilities that depend on length of strand between the compartments. (B) The TER model consists of a similar geometry, but instead of compartments the basic model units are the current loops (numbered 1–10). The outer current loop (number 11) has a voltage source (*V*_*s*_) to enable the computation of the total resistance. Resistance *R*_*ij*_ is the resistance of the strand shared by current loops *i* and *j*, except for those with *i* = *j*, when the resistor is not shared by other loops. Again, the resistances vary based on given probabilities that depend on the length of strand between the compartments. (C) Illustration of the geometrical parameters that describe the bTJ strands. *n*_*strand*_, strand number; *h*_*comp*_, small compartment height; *w*_*comp*_, small compartment width; *w*_*TJ*_ TJ half-width; *h*_*strand*_, height of single bTJ strand; *l*_*break*_, size of the break in the bTJ strand.

The computational models of the dynamic TJ structure for both the molecular permeability and TER use common geometry and dynamic parameters. Both models are divided into the bTJ and the tTJ components. The bTJ geometry is constructed as a two-dimensional structure with a uniformed depth based on the vertical model plane cutting through the TJs in the thin lateral space between the cells as shown in Fig 1B. The real TJ strand network structure is further simplified to an overlapping tile-like ordered network (Figs 1C and 2). Only a section of the strand network is modeled, but the models are averaged for the whole epithelium by long simulation times and by running them multiple times. The main assumptions of the models are as follows:

- TJs form the governing barrier against ionic and permeation of noncharged molecules in the epithelium
- Leak pathway is formed by both the static tricellular pores and by the bicellular strand dynamics, as suggested by Liang & Weber [20]
- tTJ pores are assumed to be similar between epithelia
- bTJ strands are impermeable to molecules too large to pass through claudin pores but not to ions
- bTJ strands undergo stochastic breaking and resealing events in the scale of seconds to tens of seconds
- bTJ strands have homogeneous properties throughout the network
- In the time scale of the model, molecular diffusion rate and fluid resistance inside the compartments have no effect on permeability and TER, respectively.

The bTJ molecular permeability model is based on a multi-compartmental approach. Thus, the strand dynamics and the geometry presented as an example structure in Fig 2A are incorporated into rate constants that describe the permeation between the compartments lined by the bTJ strands. The rate constants depend on a stochastic component that describes the strand state as either intact or broken, and whose value changes based on given probabilities. The amount of substance in the basal compartment below the strand network remains constant and the amount of substance in the apical compartment above the strand network is used to calculate the molecular permeability. The model is simulated using a PEG molecule with the mass of 547 Da (calculated radius of 0.51 nm), since it is unable to permeate through the claudin pores and has been used in many experimental permeability studies. The static tTJ pore pathway is combined with the bTJ results afterwards.

The bTJ resistance model uses the same geometry and dynamics as the molecular permeability model. However, the system is solved using nodal analysis and Kirchhoff’s circuit laws. An example resistor network system is shown in Fig 2B. Current loops form in the resistor network as depicted, and Kirchhoff’s loop rule is used for each loop, combined into an equation group, and solved. The dynamics and the geometry shown in Fig 2B are incorporated into the resistances between the compartments. In a similar way to the permeability model, the tTJ resistance is calculated separately and summed with the bTJ simulation results.

### Molecular permeability model

The permeabilities of the bTJ and the tTJ components are calculated separately and then combined in the end based on the parallel connection. The bTJ molecular permeability model is constructed using a multi-compartmental approach. The amount of substance in the small bTJ compartments as a function of time is described by an equation of the form

$$\begin{array}{c} \frac{\text{d}q_{i}\left(t\right)}{\text{d}t}=\sum_{j\neq i}^{n}\left(k_{ji}\left(t\right)q_{j}\left(t\right)-k_{ij}\left(t\right)q_{i}\left(t\right)\right), \end{array}$$ (1)

where *q*_*i*_(*t*) is the amount of substance in compartment *i* at time *t* and *k*_*ji*_(*t*) is the time-dependent rate constant for permeation from compartment *j* to *i* (s^–1^). Because the model is in 2D, the unit of *q*_*i*_ is m^–1^ instead of unity. This is taken into account when the results are calculated with Eq 8.

Since we consider the apical compartment to be considerably larger than the small bTJ compartments, we assume that the concentration in it remains very low. Thus, we can ignore the backflow of molecules from the apical compartment into the bTJ compartments, as shown in Fig 2A. The amount of substance in the basal compartment is assumed to remain constant (d*q*_*basal*_(*t*)/d*t* = 0).

Because we assume no molecular permeation through an intact strand, the rate constants for the permeation between the compartments depends only on the strand break permeability coefficient:

$$\begin{array}{c} k_{ij}\left(t\right)=\frac{l_{break}r_{ij}\left(t\right)}{A_{i}}P_{break}, \end{array}$$ (2)

where *l*_*break*_ is the size of the break in the strand, as indicated in Fig 2C (m), *r*_*ij*_(*t*) is a function describing the state of the strand between compartments *i* and *j*, *A*_*i*_ is the area of compartment *i* (m^2^), and *P*_*break*_ is the permeability coefficient of the strand break of indefinite length (m s^–1^). In a 3D model, break area and compartment volume would be used instead of the break size and compartment area, respectively [68].

Function *r*_*ij*_(*t*) can have a value of either 0 or 1, describing intact and broken states, respectively. The value of *r*_*ij*_(*t*) can change with given probabilities: If the strand is intact, a break will form with the probability *p*_*break*_ (m^–1^s^–1^), and if the strand is broken, it will seal with the probability *p*_*seal*_ (s^–1^). To obtain the break forming probability for a section of strand between two compartments, *p*_*break*_ must be multiplied by the length of that section. This means that longer sections of strand have higher value of *p*_*break*_. Based on the time scale of the dynamics, the possible changes in the strand states were chosen to happen every second.

The initial state of the strands can be either intact or broken. The probability of them being in either state is calculated with Markov chain after infinite time:

$$\begin{array}{c} {}[\begin{array}{cc} p_{ij,intact} & p_{ij,broken} \end{array}]=[\begin{array}{cc} \frac{1}{2} & \frac{1}{2} \end{array}][\begin{array}{cc} 1-p_{break}l_{ij} & p_{break}l_{ij}\\ p_{seal} & 1-p_{seal} \end{array}{]}^{\infty }, \end{array}$$ (3)

where *p*_*ij*,*intact*_ and *p*_*ij*,*broken*_ are the probabilities of the strand section between compartments *i* and *j* being in intact or broken states initially, respectively, and *l*_*ij*_ is the length of the strand section between those compartments (m).

Since the break size is taken into account in Eq 2, *P*_*break*_ is calculated relative to the amount of TJs in the entire epithelium, and thus making it dependent on the cell boundary length per area of epithelium:

$$\begin{array}{c} P_{break}=\frac{\epsilon_{TJ}D_{0}H_{s}\left(r_{m}/w_{TJ}\right)}{h_{strand}}, \end{array}$$ (4)

where *ϵ*_*TJ*_ is the relative area of the TJs in the epithelium, *D*_0_ is the aqueous diffusion coefficient of the permeating molecule (m^2^ s^–1^), *H*_*s*_(*r*_*m*_/*w*_*TJ*_) is the break slit hindrance factor that depends on the molecular radius *r*_*m*_ (m) and on the TJ half-width *w*_*TJ*_ (m), and *h*_*strand*_ is the bTJ strand height (m) (See Fig 2C for an illustration of the geometrical parameters) [69]. Parameter *ϵ*_*TJ*_ is calculated as

$$\begin{array}{c} \epsilon_{TJ}=2w_{TJ}l_{cb}, \end{array}$$ (5)

where *l*_*cb*_ is the cell boundary length per area of epithelium (m^–1^). Function *H*_*s*_ describes how the break walls affect the diffusion of a molecule of a given size and was derived by Dechadilok & Deen [70] by fitting a polynomial to computational results as

$$\begin{array}{c} H_{s}\left(\lambda \right)=1+\frac{9}{16}\lambda \text{ln}\left(\lambda \right)-1.19358\lambda +0.4285\lambda^{3}-0.3192\lambda^{4}+0.08428\lambda^{5}, \end{array}$$ (6)

where λ = *r*_*m*_/*w*_*TJ*_.

With zero initial conditions for the bTJ compartments, there is a so-called lag phase in the beginning of a simulation, especially with systems having more horizontal strands and lower value of *p*_*break*_. During this phase, the increase in the amount of substance in the apical compartment (*q*_*apical*_) is nonlinear. Since the permeability is calculated from the linear phase in *q*_*apical*_, the simulation is made to enter straight into this phase by setting the initial values of the amount of substance in each of the small bTJ compartments to the equilibrium state during the linear phase. This is done by simulating the permeability model multiple times and for a long time with zero initial conditions to obtain the equilibrium values for each compartment row.

The constant amount of substance for the basal compartment is set to

$$\begin{array}{c} q_{basal}=c_{basal}N_{A}A_{basal}, \end{array}$$ (7)

where *c*_*basal*_ is the basal compartment concentration (M), *N*_*A*_ is Avogadro’s constant (6.022 × 10^23^ mol^–1^), and *A*_*basal*_ is the area of the basal compartment, replacing the volume since the model is in 2D (m^2^).

The system of differential equations described by Eq 1 is solved using Matlab’s (Release 2015b, The MathWorks, Inc., Natick, Massachusetts, United States) ode23 ordinary differential equation solver. This solver uses second and third order Runge-Kutta formulas, and we found that it gives the same results as the more robust fourth and fifth order Runge-Kutta solver (ode45), while being considerably faster. The simulation is run multiple times and the linear phase of the average *q*_*apical*_ curve is used to calculate the bTJ permeability coefficient:

$$\begin{array}{c} P_{bTJ}=\frac{\text{d}q_{apical}\left(t\right)}{\text{d}t}\frac{1}{w_{model}c_{basal}}, \end{array}$$ (8)

where *w*_*model*_ is the width of the model system (m), which replaces the area in this 2D model. Next, a first degree polynomial is fitted to the linear phase of the average *q*_*apical*_ to obtain the slope. Since the relative area of the junctions in the epithelium is considered in Eq 4, *P*_*bTJ*_ is already scaled for the bTJs in the whole epithelium.

The tTJ central tubes are modeled as static pores, and an equation similar to Eq 4 is used:

$$\begin{array}{c} P_{tTJ}=\frac{\epsilon_{tTJ}D_{0}H_{p}\left(r_{m}/r_{tTJ}\right)}{h_{tTJ}}, \end{array}$$ (9)

where *ϵ*_*tTJ*_ is the relative area of the tricellular pores in the epithelium, *H*_*p*_(*r*_*m*_/*r*_*tTJ*_) is pore hindrance factor, *r*_*tTJ*_ is the tricellular pore radius (m), and *h*_*tTJ*_ is the tricellular pore height (m) [69]. The relative area of the pores is calculated as

$$\begin{array}{c} \epsilon_{tTJ}=\pi r_{tTJ}^{2}\rho_{tTJ}, \end{array}$$ (10)

where *ρ*_*tTJ*_ is the density of the tricellular junctions in an epithelium (m^–2^). The equation for *H*_*p*_ was also derived by Dechadilok & Deen [70] by fitting a polynomial to computational results as

$$\begin{array}{c} \begin{array}{cc} H_{p}\left(\lambda \right)= & 1+\frac{9}{8}\lambda \text{ln}\left(\lambda \right)-1.56034\lambda +0.528155\lambda^{2}+1.91521\lambda^{3}-2.81903\lambda^{4}\\ & +0.270788\lambda^{5}+1.10115\lambda^{6}-0.435933\lambda^{7} \end{array}, \end{array}$$ (11)

where λ = *r*_*m*_/*r*_*tTJ*_. This equation is more accurate than the much-used Renkin equation when λ is close to unity [70].

The total epithelial TJ permeability is finally calculated based on the parallel connection between the two pathways as

$$\begin{array}{c} P_{TJ}=P_{bTJ}+P_{tTJ}. \end{array}$$ (12)

The required properties of the permeating molecule for this model are the molecular radius and the aqueous diffusion coefficient. Since we use a PEG oligomer with a mass of 547 Da, an equation relating the mass of a PEG oligomer to its size is used [71]:

$$\begin{array}{c} r_{m}=0.29M_{m}^{0.454}, \end{array}$$ (13)

where *r*_*m*_ is in Å (0.1 nm) and *M*_*m*_ is the molecular mass (Da). The aqueous diffusion coefficient is calculated with an empirical relationship derived by Avdeef [61]:

$$\begin{array}{c} D_{0}=9.9\times 10^{-9}M_{m}^{-0.453}. \end{array}$$ (14)

The default simulation time for the bTJ model is two hours and the stochastic behavior is further averaged by running the simulations 512 times. We found that the results were not affected by further averaging. The simulations were run using the Finnish IT Center for Science’s (CSC) Taito supercluster using parallel computing (nodes with two 12-core Intel Haswell E5-2690v3 processors running at 2.6 GHz and 128 GB of DRR4 memory operating at 2133 MHz). A Matlab implementation of the permeability model is given in S1 File.

### TER model

In the TER model, the bTJ and tTJ components are again calculated separately and connected in parallel in the end. The bTJ resistance model is constructed as a network of dynamic resistors (Fig 2B). Since we are interested in the resistance, the strand capacitance is ignored. For each current loop *i* (Fig 2B), the equation is of the form

$$\begin{array}{c} \sum_{j}^{n}R_{ij}\left(t\right)I_{i}-\sum_{i\neq j}^{n}R_{ij}\left(t\right)I_{j}=\{\begin{array}{cc} V_{s}, & \text{for}\;\text{the}\;\text{outer}\;\text{current}\;\text{loop}\;\\ 0, & \text{otherwise} \end{array}, \end{array}$$ (15)

where *R*_*ij*_(*t*) is the time-dependent resistance of the section of strand that is shared by current loops *i* and *j* (Ω), *I*_*i*_ is the current in loop *i* (A), and *V*_*s*_ is the voltage applied by the external source in the outer loop (V).

The strand dynamics are incorporated into the resistances *R*_*ij*_(*t*), making them analogous to the rate constants in the bTJ permeability model. Because ions can also pass through the intact strands, *R*_*ij*_(*t*) depends on both the intact strand and break resistances:

$$\begin{array}{c} R_{ij}\left(t\right)=(\frac{\left(l_{ij}-l_{break}\right)r_{ij}\left(t\right)}{R_{strand}}+\frac{r_{ij}\left(t\right)}{R_{break}}{)}^{-1}, \end{array}$$ (16)

where *l*_*ij*_, *l*_*break*_, and *r*_*ij*_(*t*) have been described in the bTJ permeability model, *R*_*strand*_ is the intact strand resistance per strand length (Ωm), and *R*_*break*_ is the resistance of a break (Ω).

The break resistance is calculated with the equation

$$\begin{array}{c} R_{break}=\frac{\rho_{em}h_{strand}}{A_{break}}, \end{array}$$ (17)

where *ρ*_*em*_ is the resistivity of the extracellular medium (Ωm) and *A*_*break*_ is the area of the break in the strand (m^2^), calculated as 2*w*_*TJ*_ *l*_*break*_.

Although the TER measurement is basically instantaneous, the bTJ resistance model is simulated for a long time to average the results. The current flowing in the outer loop *I*_*outer*_ (A) is used to calculate the bTJ resistance at each time point with Ohm’s law:

$$\begin{array}{c} R_{bTJ}\left(t\right)=\frac{V_{s}}{I_{outer}\left(t\right)}\frac{w_{model}}{l_{cb}}, \end{array}$$ (18)

where the factor *w*_*model*_/*l*_*cb*_ scales the results for the whole epithelium. To solve the bTJ model, the linear system defined by Eq 15 is transformed to matrix form and solved using Matlab.

The pores in the tTJ tubes are modeled as a static and their resistance is calculated as

$$\begin{array}{c} R_{tTJ}=\frac{\rho_{em}h_{tTJ}}{\pi {r_{tTJ}}^{2}\rho_{tTJ}}. \end{array}$$ (19)

For each simulation time point of the bTJ resistance model, the total TER is calculated based on the parallel connection as

$$\begin{array}{c} TER\left(t\right)=(\frac{1}{R_{bTJ}\left(t\right)}+\frac{1}{R_{tTJ}}{)}^{-1}. \end{array}$$ (20)

Finally, to obtain the average TER for the simulation, the time average is taken from the results. The simulation time is 10^6^ seconds and the simulations were run using the CSC’s Taito supercluster with serial computing. A Matlab implementation of the TER model is given in S2 File.

### Parameter values

Here we describe the default values of the model parameters. The TJ structure in our model is described by bTJ compartment dimensions, strand number (*n*_*strand*_), strand height (*h*_*strand*_), TJ half-width (*w*_*TJ*_), tricellular pore radius (*r*_*tTJ*_), and tricellular pore height (*h*_*tTJ*_). The TJ dynamics are described by break forming and sealing probabilities (*p*_*break*_ and *p*_*seal*_, respectively), and break size (*l*_*break*_).

Although there is a lot a variety in the TJ strand morphology [5, 72–75], we used one strand morphology since the main focus is the break dynamics. The chosen bTJ compartment width (*w*_*comp*_) and height (*h*_*comp*_) were both 100 nm. These values are in the range of the bTJ compartment sizes found in the freeze-fracture replicas [5, 72–75] as well as by Kaufmann et al. using super-resolution microscopy [76]. The horizontal number of the compartments in the simulated systems was set to 50 and the heights of the apical and basal compartments in the molecular permeability model were both set to 200 nm. Based on the strand numbers in MDCK monolayers (3–5 strands) [52, 53, 74, 77], Caco-2 monolayers (4–5 strands) [78], and retinal pigment epithelium (4 strands) [39], the default strand number was set to *n*_*strand*_ = 4.

The value of *h*_*strand*_ = 6 nm was based on the electron microscopy of TJ freeze-fracture replicas and the TJ strand architecture model by Suzuki et al. [14]. TJ half-width was chosen as *w*_*TJ*_ = 4 nm, estimated based on the architecture model by Suzuki et al. [14] and transmission electron microscope images [54, 79, 80]. The dimensions of the tricellular pores, *r*_*tTJ*_ = 5 nm and *h*_*tTJ*_ = 1 *μ*m, were taken from the measured values from freeze-fracture replicas [7, 16].

The dynamic parameters of the strand dynamics were more uncertain due to the lack of experimental data. The strand breaks were assumed to remain open on average around 30 seconds based on the time scale of the dynamics in the transfected fibroblasts [46, 50]. This led to the break sealing probability of *p*_*seal*_ = 0.033 s^–1^. The break size was approximated based on the figures and videos by Sasaki et al. [46] and by Van Itallie et al. [50], leading to a value of *l*_*break*_ = 20 nm. This value was also used to quantify breaks by Rosenthal and coworkers [52, 53]. The break forming probability (*p*_*break*_) is fitted in the Results section based on the literature data. Due to the time scale of the dynamics and computational limitations, we restricted the possible state changes in the strands to occur every second.

The basal compartment concentration and the voltage of the external source used to measure the resistance are scaling parameters and do not affect the results. The chosen values were *c*_*basal*_ = 1 mM and *V*_*s*_ = 1 V, respectively. Also, the resistive properties of the breaks and the strands are needed. The resistivity of the extracellular medium required for the breaks was *ρ*_*em*_ = 0.537 Ωm [16]. The value of the strand resistance (*R*_*strand*_) depends on the epithelium, and is fitted in the Results section.

The cell boundary length per epithelial area (*l*_*cb*_) and tTJ pore density (*ρ*_*tTJ*_) are also highly dependent on the epithelium. They were determined using ImageJ Fiji [81, 82] from the immunofluorescence microscopy images illustrating the cell-cell junctions in the studies our models were fitted for, and the values are described in the Results section. The only unknown parameters values were *p*_*break*_ (both permeability and TER models) and *R*_*strand*_ (TER model). These values are found by iteratively fitting the models to the experimental data. The model parameters described here are summarized in Table 1.

**Table 1:** Model parameters.

Description	Parameter	Value	Reference
bTJ compartment width	*w*_*comp*_	100 nm	^a^
bTJ compartment height	*h*_*comp*_	100 nm	^a^
bTJ strand number	*n*_*strand*_	4	[39, 52, 53, 77, 78]
Single bTJ strand height	*h*_*strand*_	6 nm	[14]
TJ half-width	*w*_*TJ*_	4 nm	^a^
tTJ pore radius	*r*_*tTJ*_	5 nm	[7]
tTJ pore height	*h*_*tTJ*_	1 *μ*m	[7, 16]
Break sealing probability	*p*_*seal*_	0.033 s^–1^	^a^
Break size	*l*_*break*_	20 nm	^a^
Basal compartment concentration	*c*_*basal*_	1 mM	^a^
Voltage source in the external loop	*V*_*s*_	1 V	^a^
Extracellular medium resistivity	*ρ*_*em*_	0.537 Ω m	[16]

^a^ See text for explanation.

## Results

### Model fitting and the origin of the leak pathway

The models were used to study the roles of tricellular junctions and bicellular strand dynamics in the leak pathway for the epithelial molecular permeability and TER. The models were fitted to the experimental data by varying the values of the break forming probability (*p*_*break*_) and the TJ strand resistance (*R*_*strand*_). Since the cell boundary length (*l*_*cb*_) and tTJ density (*ρ*_*tTJ*_) had a strong impact on the simulation results (see Parameter sensitivity analysis), we only used experimental data that included immunofluorescence microscopy images showing the cell-cell junctions. Therefore, unfortunately, the PEG profiling studies by Watson et al. [31, 32], Van Itallie et al. [35], and Linnankoski et al. [36] had to be excluded from our model fitting.

First, the permeability model was fitted to the experimental data of 547-Da PEG oligomer permeation, since this molecule utilizes the leak pathway and it was used in the suitable studies [33, 34, 54, 83]. The fitting was done by iteratively changing the value of *p*_*break*_ (with the accuracy of 0.001 *μ*m^–1^ s^–1^) and comparing the simulation result with the experimental result. The value of *p*_*break*_ for MDCK C7 was calculated rather than fitted since the tTJ pores were enough to form the leak pathway for this epithelium, and thus making the fitting impossible. The value was iteratively calculated with Eq 3 using the chosen break sealing probability and the average amount of breaks per strand length for high-TER MDCK [52, 53]. Next, the TER model was fitted using the obtained values of *p*_*break*_ to iteratively find the values of *R*_*strand*_ (with the accuracy of 0.01 GΩ *μ*m). The experimental data used to fit the model, the values of experimental permeability and TER, the values of *l*_*cb*_ and *ρ*_*tTJ*_, as well as the fitting results are shown in Table 2.

**Table 2:** Values of the model parameters used to fit the molecular permeability and the TER model.

Epithelia	*P*_*exp*_	*TER*_*exp*_	*l*_*cb*_	*ρ*_*tTJ*_	*p*_*break*_	*R*_*strand*_
	(nm s^–1^)	(Ω cm^2^)	(*μ*m^–1^)	(*μ*m^–2^)	(*μ*m^–1^ s^–1^)	(GΩ *μ*m)
Caco-2 [33]	10.0	220	0.525	0.130	0.047	7.65
MDCK C7 [33]	1.0	460	0.424	0.078	0.005*	10.62
MDCK IIa [33]	4.3	28	0.484	0.106	0.032	0.46
MDCK IIb [34]	0.8	54	0.185	0.014	0.029	0.32
MDCK IIc [83]	2.9	41	0.311	0.035	0.038	0.45
MDCK IId [54]	2.3	30	0.179	0.014	0.044	0.20
MDCK IIb ZO-1 KD [34]	3.1	62	0.189	0.015	0.047	0.45
MDCK IId ZO-1/2 dKD [54]	26.0	26	0.200	0.019	0.084	0.34

*P*_*exp*_, experimental permeability of 547-Da PEG; *TER*_*exp*_, experimental TER; *l*_*cb*_, cell boundary length per area; *ρ*_*tTJ*_, tricellular TJ pore density; *p*_*break*_, break forming probability; *R*_*strand*_, strand resistance.

* calculated.

The cell sizes in MDCK II monolayers, as indicated by the cell boundary length per area (*l*_*cb*_) and and the tricellular pore density (*ρ*_*tTJ*_) in Table 2, varied greatly between the measurements. However, they were on average the largest of the fitted epithelia. The cells in Caco-2 monolayer were the smallest and in MDCK C7 monolayer between the two extremes. Surprisingly, the experimental permeability of high-resistance MDCK C7 was higher than that of the low-resistance MDCK IIb, which is most likely explained by the difference in cell size.

Although there was some variation in the values of *p*_*break*_ for MDCK II, they are similar to each other having a mean (±SD) of 0.036 (±0.007). This is especially interesting when considering the great variability in the cell size. As for the TER, the variation was higher, with a mean (±SD) of 0.36 (±0.12) GΩ *μ*m. The values of both *p*_*break*_ and *R*_*strand*_ were found to differ significantly for MDCK C7. Its values of *p*_*break*_ and *R*_*strand*_ were 7.2 times lower and over 30 times larger, respectively, compared with those of the average MDCK II. The properties of Caco-2 were a combination of the two MDCK strains: While the value of *p*_*break*_ was similar to that of the MDCK II, *R*_*strand*_ was closer to the value of MDCK C7. The resistance of a single 20-nm break in the strands was *R*_*break*_ = 0.2 GΩ. The resistances of the same length of strand for average MDCK II, MDCK C7, and Caco-2 were 18 GΩ, 531 GΩ, and 383 GΩ, respectively. Thus, the strands had 90 to 2670 times higher resistance compared with the breaks.

To further check the validity of our results, we used the MDCK C7 *p*_*break*_ and *R*_*strand*_ values to simulate the TER for the original MDCK C7 epithelia (measured TER 5650 Ω cm^2^) [84] by changing the cell size. Unlike with the other results considered here, the figures in [84] did not allow rigorous determination of the cell size properties, and therefore the cell radius was estimated to be between 15 and 20 *μ*m (Fig 1A in [84]). Assuming perfect hexagonal cell array, we calculated the values of *l*_*cb*_ and *ρ*_*tTJ*_ to be between 0.050–0.067 *μ*m^–1^ and 0.001–0.003 *μ*m^–2^, respectively. The obtained TER values for these cell radii of 15 and 20 *μ*m were 4820 and 7310 Ω cm^2^, respectively. This indicated that the difference in cell size explains the difference in TER between the two experimental MDCK C7 results.

**Fig 3:**
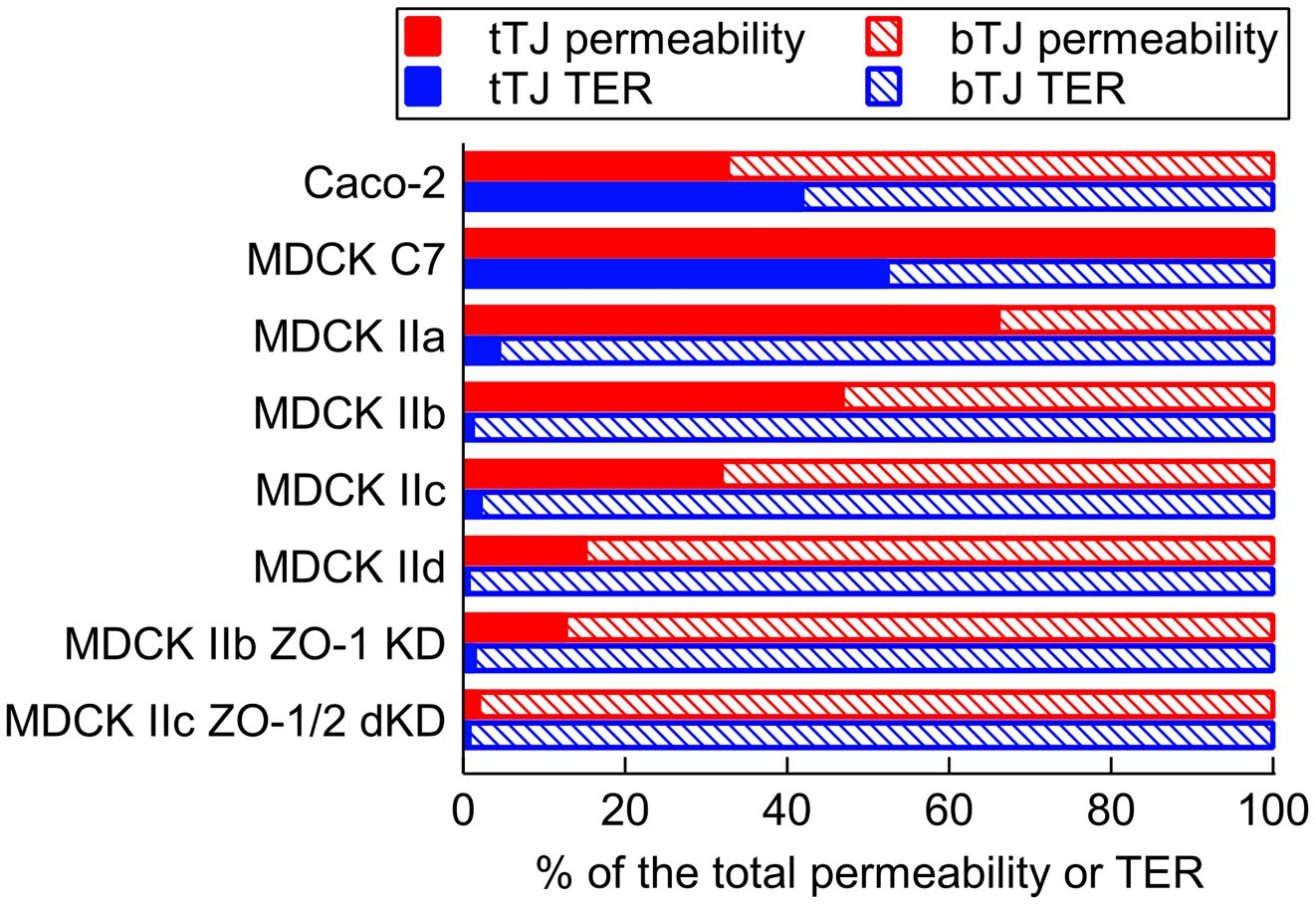
Relative roles of the tricellular and bicellular pathways. The relative roles of tTJ (solid) and bTJ (dashed) on both the molecular permeability (red) and TER (blue) for all the simulated epithelia (Caco-2 [33], MDCK C7 [33], MDCK IIa [33], MDCK IIb [34], MDCK IIc [83], MDCK IId [54], MDCK IIb ZO-1 KD [34], and MDCK IIc ZO-1/2 dKD MDCK IId [54]).

Fig 3 shows the relative contributions of the two assumed leak pathway components—the tTJ pores and the bTJ strand dynamics—to the total leak pathway. MDCK C7 was the only epithelia considered here whose permeability was dominated by the tTJ pathway. Since the leak pathway was originally defined by the permeability, it can be said that the MDCK C7 leak pathway was fully formed by the tTJ pores. The role of bTJ dynamics was more prevalent, but variable, for the MDCK II permeability, having a mean (±SD) of 60.1 (±21.7)%. The bTJ dynamics was also the main pathway for the Caco-2 permeability. As for the TER, the role of the tTJ pores was insignificant for the MDCK II with a mean (±SD) relative role of 2.0 (±1.7)% between the four measurements. In both MDCK C7 and Caco-2, the tTJ pathway formed approximately half of the resistance of the epithelium.

### Simulating experimentally-induced changes in the TJs

Next, we investigated how the developed models can recapitulate disturbances or changes in the TJ proteins, based on the studies of ZO-1 knockdown in MDCK II by Van Itallie et al. [34] and double ZO-1/2 knockdown in MDCK II by Fanning et al. [54]. Since both suggested that the observed changes in the barrier properties caused by these knockdowns were a result of decreased strand stability, we fitted our models to these results by varying the break forming probability *p*_*break*_. The TER model also required fitting of *R*_*strand*_. The results of the fitting are shown in Table 2 and the relative pathway roles in Fig 3 before (MDCK IIb and MDCK IIc) and after (MDCK IIb ZO-1 KD and MDCK IIc ZO-1/2 dKD) the knockdowns.

According to our results, the knockdown of ZO-1 led to a 62% increase in *p*_*break*_ and to a 41% increase in *R*_*strand*_. In addition, the decreased bTJ tightness resulted in a 65% increase in the relative role of the bTJ pathway. The effect of the ZO-1/2 double knockdown was larger; it caused a 121% increase in *p*_*break*_ and a 70% increase in *R*_*strand*_. The increase in the relative role of bTJ caused by the double knockdown (44%) was smaller than that of the ZO-1 knockdown due to the higher original contribution of bTJ in MDCK IIc. The changes in the relative roles of the pathways in TER were insignificant for both the single and double knockdown.

### The effect of strand number on permeability and TER

**Fig 4:**
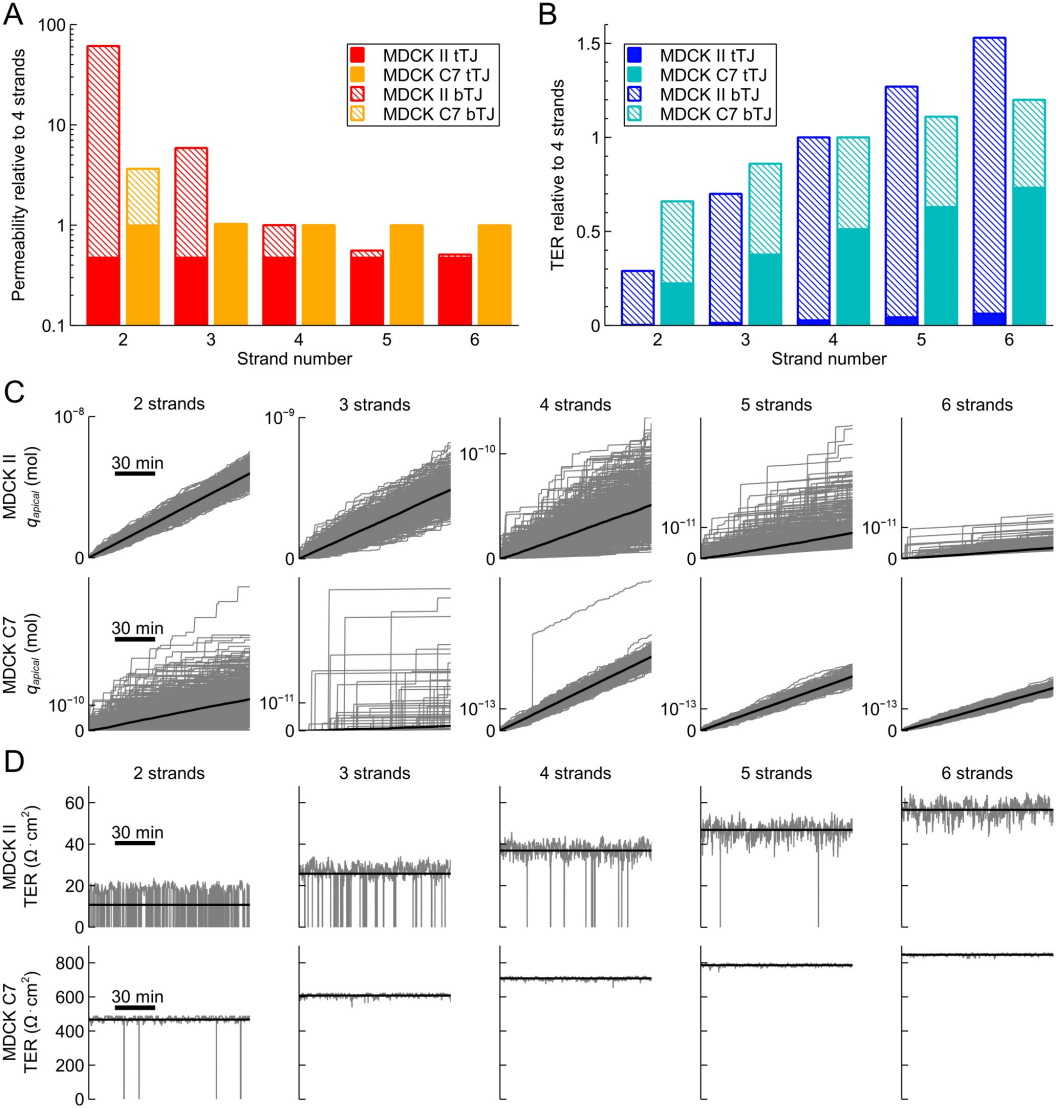
Effect of strand number on permeability and TER. The effect of strand number (*n*_*strand*_ = [2, 6]) on (A) molecular permeability and (B) TER in average MDCK II (red and blue) and MDCK C7 (orange and cyan), shown relative to the system with 4 strands. The relative roles of tTJ (solid) and bTJ (dashed) pathways are also illustrated relative to the 4-strand total values. (C) Raw simulation data of the 512 simulations of the apical amount of substance (*q*_*apical*_) as a function of time for average MDCK II (top) and MDCK C7 (bottom) for systems with the strand number from 2 to 6. The average values of the 512 for each time point are shown with the black lines. (D) TER as a function of time for average MDCK II (top) and MDCK C7 (bottom) during a 2-hour section of the simulation for systems with the strand number from 2 to 6. The average values for the whole simulation are shown with the black lines.

Next, we studied the effect of the number of strands on the barrier properties by changing the strand number (*n*_*strand*_) for the average MDCK II and MDCK C7 epithelia. These monolayers were chosen to illustrate the effect of *n*_*strand*_ for different levels of strand dynamics and strand resistances. Although these epithelia do not necessarily manifest varying numbers of strands, they provide two systems with different properties to base our simulations on. We simulated the model with *n*_*strand*_ = [2, 6] for both permeability and TER, and the results for these simulations are shown in Fig 4A and 4B, respectively. To remove the impact of the cell size from the comparison, the simulations were run with the mean values of cell boundary length per area (*l*_*cb*_ = 0.282 *μ*m^–1^) and tricellular TJ pore density (*ρ*_*tTJ*_ = 0.049 *μ*m^–2^) of all the MDCK data included here (2 and C7).

Naturally, the permeability decreased as *n*_*strand*_ increased (Fig 4A). In addition, the increase in *n*_*strand*_ also led to an increase in the relative role of the tTJ pathway of the total permeability. This growing importance of the tTJ pathway led to saturation of the permeability at approximately 6 and 3 strands for MDCK II and C7, respectively. In contrast, TER grew when *n*_*strand*_ increased (Fig 4B). However, similarly to permeability, the significance of the tTJ pores increased with *n*_*strand*_. TER also showed the saturation behavior, but the saturation occurred past the simulated strand numbers for both of the MDCK strains. Moreover, the scale of the changes caused by the varying *n*_*strand*_ were considerable larger for MDCK II in both permeability and TER. Also, the largest difference in permeability relative to the 4-strand standard system was almost two orders of magnitude compared with the under one order of magnitude for TER.

The raw, unaveraged simulation data indicating the time course of the simulations (Fig 4C and 4D) showed the different behavior in the MDCK II and C7 monolayers for both permeability and TER. Full opening events, in which there was an open pathway through the strand network via the breaks, are indicated in the TER results by the sharp downward spikes. The spikes disappeared altogether at 6 strands for MDCK II and at 3 strands for MDCK C7. These events were not always clear in the permeability results, since the sharp steps in *q*_*apical*_ may be a result of molecules stored into the small compartments released into the apical compartment. For example, as indicated by the TER results of 2-strand MDCK II, there was at least one full opening present around half of the time. However, no sharp steps were seen in *q*_*apical*_ in any of the simulations shown. In contrast, there were multiple, minuscule scale steps in *q*_*apical*_ e.g. for 6-strand MDCK II.

As *n*_*strand*_ increased, the slopes for the bulk of the permeability simulations decreased for both MDCK II and C7 (Fig 4C and 4D). However, the increase in *n*_*strand*_ in MDCK II led to more variable simulation results as well as to an increase in the number of the visibly different *q*_*apical*_ curves with full openings. Interestingly, the behavior of MDCK C7 was different; generally, when *n*_*strand*_ increased, the variability in the results decreased. This was most likely due to the lack of full opening events. Furthermore, the clearly distinct *q*_*apical*_ curves in the 3-strand MDCK C7 differed considerably more from the bulk of the simulations compared with other simulated systems.

Thus, the simulation raw data showed a biphasic behavior for bTJ permeability as a function of TJ tightness, defined by both the strand number and the level of strand dynamics. With low strand numbers and high values of break forming probability, the bulk of permeability through the strand network passed via the full opening events. This resulted in low variance between the individual simulations, as shown, e.g., by the 2-strand MDCK II (Fig 4C). With high strand numbers and low values of levels of strand dynamics, however, the bulk of the permeability occurred via the step-by-step diffusion through the compartment network. Again, this resulted in low variance between the individual simulations, as shown, e.g., by the 5- and 6-strand MDCK C7 (Fig 4C). Between these two extremes, there was a transition zone where the variance between the simulations was higher.

**Fig 5:**
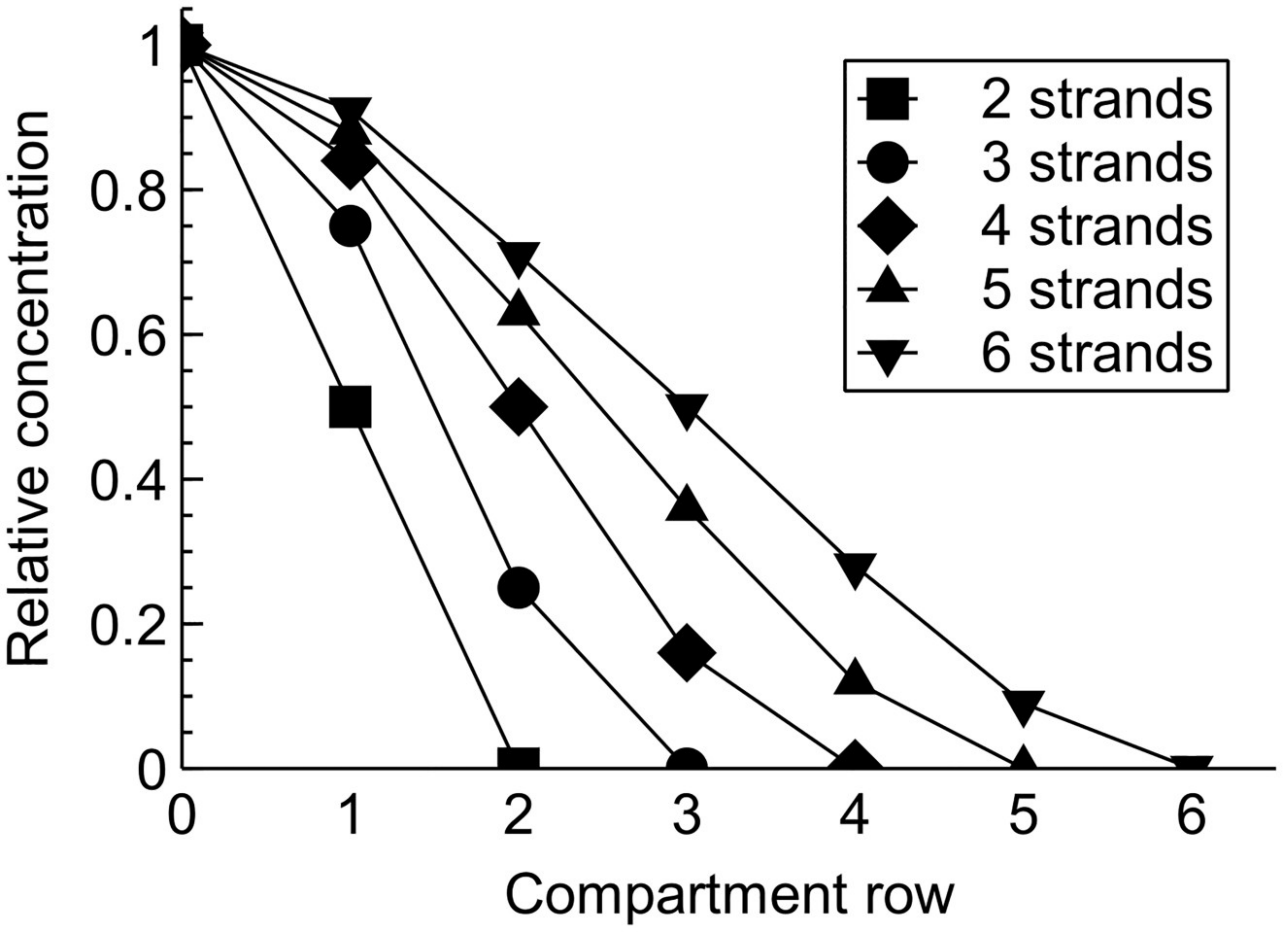
Relative equilibrium concentrations in the equilibrium state. The relative equilibrium concentrations are shown in relation to the concentration in the basal compartment (compartment row 0) for the 2–6 strand TJ systems. The compartment row with relative concentration of 0 refers to the apical compartment for that system, since its concentrations was assumed to remain very low during the simulations.

The permeability model was simulated with the equilibrium state of the system as the initial condition. These states for each of the compartment rows were found by running the models with zero initial conditions for a long time. The relative equilibrium concentrations compared to the basal compartment are shown in Fig 5 for systems with 2–6 strands. The parameters affecting the rate constants or the magnitude of *p*_*break*_ had no effect on these values; they only defined how fast the linear phase was reached. Interestingly, while the differences between the small TJ compartments were approximately linear, the equilibrium concentration change between the basal compartment and the bottom small compartment row as well as the top small compartment row and the apical compartment showed nonlinear discontinuities.

In addition, although the lag phase was not included in the simulations, we calculated the length of this phase for each of the bTJ permeability simulations to describe the time it takes for the permeating molecules to pass through the TJs. This was done by running the simulation with zero initial values for the small bTJ compartments and by extrapolating the linear phase of *q*_*apical*_ backwards to determine its intersection with the time axis. Longer than normal simulation times were used in some cases to obtain a linear phase of sufficient length. The lag times for both of the MDCK strains are shown in Table 3. As expected, the lag time grew as the strand number increased and as the break forming probability decreased. Interestingly, the lag time for 5-strand MDCK II and 2-strand MDCK C7 were close to each other, although having a large difference in the actual permeability coefficients. The same biphasic behavior can be seen in the lag times when the barrier became tighter. The lag times grew slowly for MDCK II as *n*_*strand*_ was increased. However, with the higher strand numbers for MDCK II and for all the results for MDCK C7, the increase in *n*_*strand*_ led to considerably larger changes in the lag times.

**Table 3:** The lag times in minutes for MDCK II and MDCK C7 with the different strand numbers.

*n*_*strand*_	MDCK II	MDCK C7
2	0.04	4.82
3	0.64	14.24
4	2.81	83.10
5	4.38	156.99
6	11.54	251.90

*n*_*strand*_, strand number.

### Comparison with steady-state models

**Fig 6:**
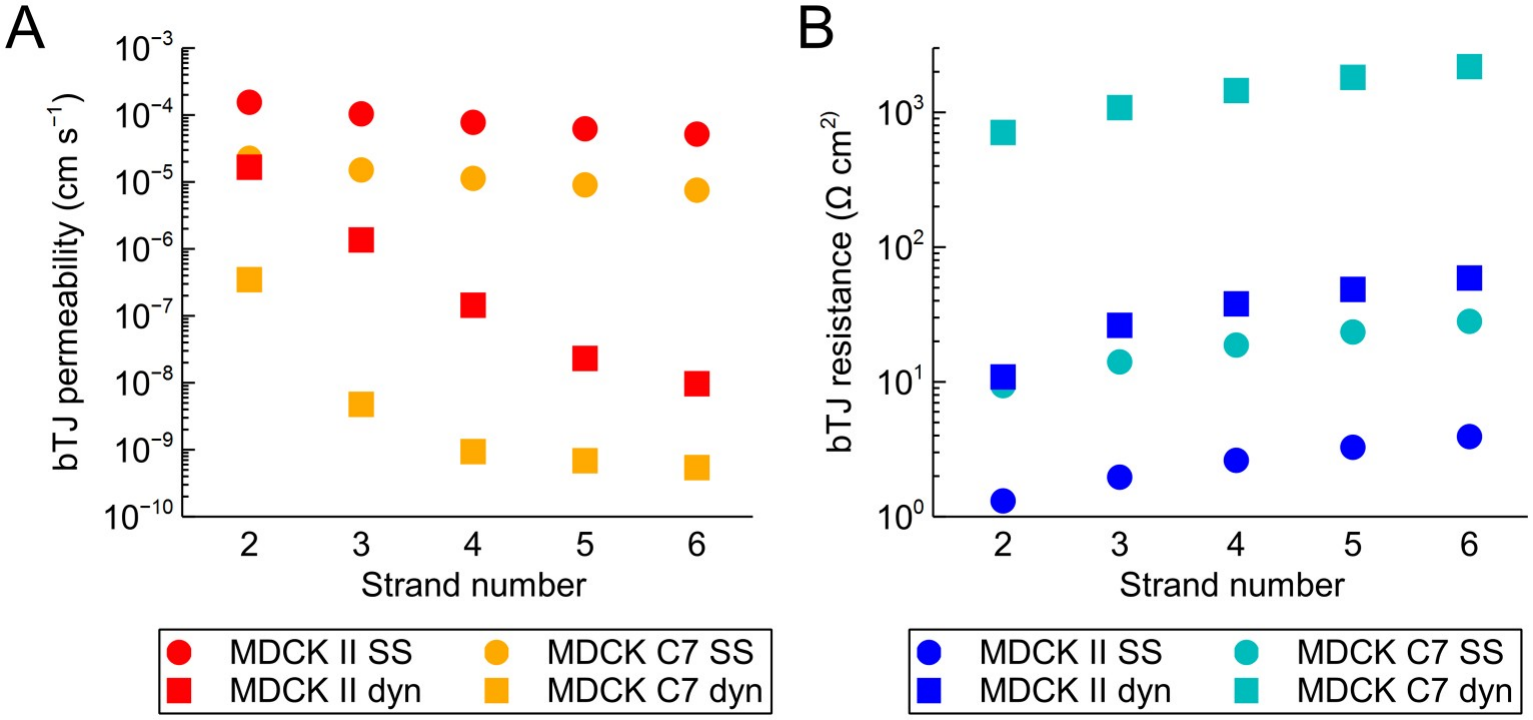
Comparison between the dynamic and steady-state models. Comparison between the bTJ results of our dynamic (dyn) model (squares) and a simple steady-state (SS) model (circles) for (A) permeability and (B) resistance. The simulations were run both for MDCK II (red and blue) and C7 (orange and cyan).

To test if the results produced by the dynamic bTJ models presented here could be reached with simpler methods, we created steady-state (SS) bTJ models that assumed a static system with an average number of breaks per strand for both barrier properties. In the SS bTJ permeability model, the compartment rows were reduced into a single large compartment between the strands, since the compartments in the same row could be assumed to be in equilibrium. The SS bTJ resistance model similarly assumed to only contain horizontal strands, and thus simplifying the model to a series connection of identical strands. The number of static open breaks for both SS models was defined from Eq 3. For comparison, we ran the simulations for the varying strand number for both MDCK II and C7 presented in the previous section, and the results of the comparison are shown in Fig 6.

It can be clearly seen that the SS models were not able to produce comparable results for bTJ permeability nor for bTJ resistance. The permeabilities predicted by the SS model were well above those produced by our dynamic model (Fig 6A). Moreover, the SS permeabilities showed very little change overall when the strand number was increased from 2 to 6. As for the bTJ resistance, the values produced by the SS model were approximately one and two orders of magnitude lower, respectively, than those from our dynamic model for MDCK II and C7 (Fig 6B).

### Parameter sensitivity analysis

**Fig 7:**
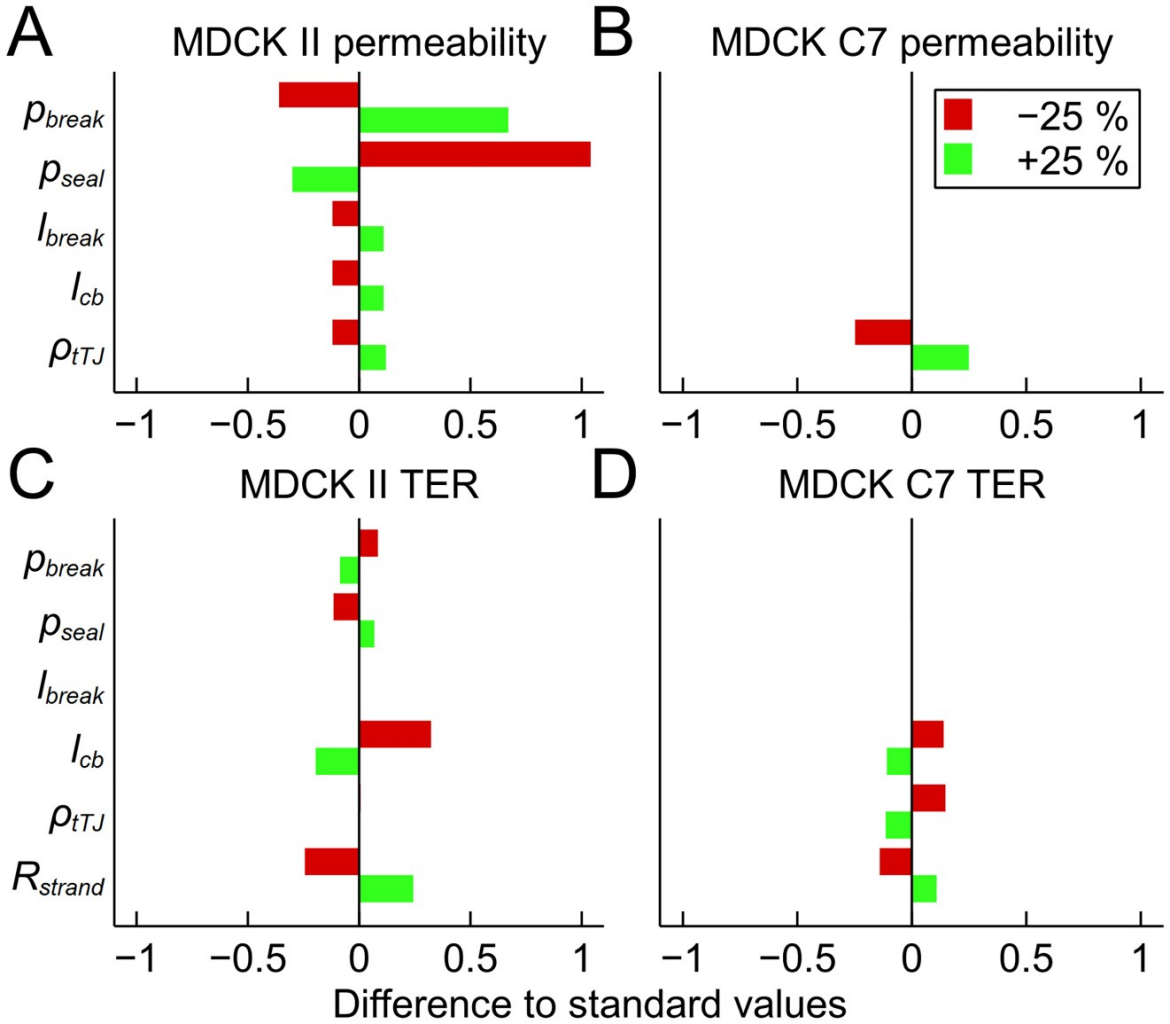
Parameter sensitivity analysis. Results of the parameter sensitivity analysis. We varied the values of the chosen parameters by ±25% and both the permeability and TER simulation results were compared with the normal system. The analysis was conducted for average MDCK II permeability (A) and TER (C) as well as MDCK C7 permeability (B) and TER (D). *p*_*break*_, break forming probability; *p*_*seal*_, break sealing probability; *l*_*break*_, break size; *l*_*cb*_, strand length per area; *ρ*_*tTJ*_, tricellular TJ pore density; *R*_*strand*_, strand resistance.

Finally, we performed a sensitivity analysis on certain model parameters by individually altering their values ±25%. The chosen parameters for both models were break forming and sealing probabilities (*p*_*break*_ and *p*_*seal*_, respectively), break size (*l*_*break*_), cell boundary length per area (*l*_*cb*_), and tTJ pore density (*ρ*_*tTJ*_). Strand resistance (*R*_*strand*_) was additionally included for the TER model. Parameters *l*_*cb*_ and *ρ*_*tTJ*_ both depend on the cell size, and thus are not independent from each other. However, by changing them individually we could observe the relative roles of these parameters. We ran the analysis for both the average MDCK II and the MDCK C7 using the same default values of *l*_*cb*_ (0.282 *μ*m^–1^) and *ρ*_*tTJ*_ (0.049 *μ*m^–2^) as with the strand number simulations. The results comparing the sensitivity simulations with the standard simulations are shown in Fig 7.

The permeability of MDCK II was very sensitive to the changes in the strand dynamics, since alterations in the break probabilities led to large changes in the permeability. The variance of these parameters had a significantly smaller effect on the MDCK II TER. While the alterations in *l*_*break*_ affected the MDCK II permeability, they had no effect on TER. Alterations in the values of *l*_*cb*_ and *ρ*_*tTJ*_ had similar levels of influence on the MDCK II permeability. The MDCK II TER was unchanged by the variance of *ρ*_*tTJ*_. However, it was affected more by the variance of *l*_*cb*_ than the permeability. Finally, the changes in TER were equal to the alterations in *R*_*strand*_ for MDCK II, indicating direct proportionality.

The results were extremely different for MDCK C7. Due to the lower level of strand dynamics, the alterations in the parameters describing the breaks (*p*_*break*_, *p*_*seal*_, and *l*_*break*_) had no effect on either permeability or TER. This was also the case with *l*_*cb*_ for permeability. On the other hand, the results indicate direct dependence of permeability on *ρ*_*tTJ*_ for MDCK C7. The impacts of both *l*_*cb*_ and *ρ*_*tTJ*_ were similar for TER, but smaller than the parameter value variations. Finally, the influence of *R*_*strand*_ for MDCK C7 was smaller than for MDCK II.

## Discussion

Tight junctions (TJs) are an indispensable part of the epithelia that form the barriers between many of the body’s compartments, and yet not enough is known about their structure or structural dynamics. In this work, we have developed a computational model of the dynamic TJ structure to study the origin and the properties of the leak pathway—the nonspecific permeation pathway through the TJs. This was done by simulating the epithelial molecular permeability of a PEG oligomer and transepithelial electrical resistance (TER) with the same structural strand dynamics for different epithelial monolayers and scenarios. The model combines the current knowledge and theory of the dynamic TJ structure into a computational framework.

There are experimental findings that attest to both candidates for the leak pathway: the tricellular junction pores and the bicellular strand dynamics. Krug et al. [16] observed that 3-kDa dextran mainly diffuses through the tTJ pores, and through the bTJs to a lesser extent. While our model did not extend to macromolecule permeation, the limited size of the strand breaks would result in the tTJ pores being the main pathway for the molecules of this size. Krug et al. also calculated the role of the tTJ pores to be minuscule for ion permeation due to their rarity [16]. At the moment, there is no direct evidence of the dynamic bTJ strand breaking and sealing events forming the leak pathway, but it has been theorized [20, 22, 33, 47, 48]. Although dynamic strand breaks have been observed in transfected fibroblasts [46, 50] and static breaks in freeze-fracture images [16, 51–53], no strand breaks were detected by Weber et al. in their bTJ patch clamp measurements [56]. They discussed multiple reasons for this, including that the breaks may not be distinguishable from the patch seal break [56]. It is, however, also possible that the strong electrode seal between the pipette and the junctional membrane might stabilize the strands mechanically and therefore prevent the strand level dynamics.

While one of the assumptions in our models was that the leak pathway is formed by both the bTJ strand dynamics and tTJ pores, interestingly, the permeability leak pathway of the MDCK C7 was formed solely by the tTJ pores. Whereas the relative roles of two pathways in permeability varied greatly between our four MDCK II fitting results, they all showed that the tTJ pores by themselves were incapable of producing the measured permeability values. A possible cause for the variation between the MDCK II results is the cell culture times in the experiments. The reported respective culturing periods for the MDCK IIa, IIb, and IId were from 4 to 8 days, from 7 to 10 days, and 10 days [33, 34, 54]. There was no culture time directly reported for MDCK IIc [83]. This indicates that longer culturing periods might have led to a higher significance of the bTJ pathway and to a higher level of strand dynamics. However, since the amount of the data is limited, this might be a coincidence. Similar to MDCK II, approximately half of the permeability leak pathway was formed by the bTJ dynamics for Caco-2. As for the TER, the MDCK II was dominated by the bTJ pathway, as was also calculated by Krug et al. [16]. On the contrary, the impact of tTJ pores on the Caco-2 and the MDCK C7 was significant, due to the higher strand resistance.

Thus, as theorized by Liang & Weber [20], our model suggests that the tTJ pores and the bTJ strand dynamics may both contribute to the leak pathway with varying degrees. Moreover, the significance of these two alternatives was different for permeability and TER. The tTJ pores were the prominent permeation pathway only for epithelia with more stable strands, since the extremely rare breaks did not enable fast step-by-step permeation. The strand dynamics had a lesser role in determining the bTJ resistance, as the strand resistance, and thus their molecular composition was the dominating factor. The tTJ pores became more important only with the higher strand resistances. This two-component leak pathway is further supported by how changes in the expression of occludin and tricellulin, effect the macromolecular permeation. Although it is not completely understood how, the expression of levels of occludin, mainly located in the bTJ strands, have been shown to regulate the leak pathway permeability [45, 83, 85, 86]. On the other hand, Krug et al. [16, 87] have shown that increased expression of tricellulin leads to a decreased permeability of >4-kDa dextrans and vice versa.

Although there was diversity in the cell sizes, the main differences between the epithelial barriers rose from the distinct levels of the bTJ strand dynamics and strand resistances. Of the epithelia considered here, Caco-2 was found to have the most dynamic strands. The obtained break forming probabilities for the MDCK II results were quite similar to each other, while MDCK C7 had extremely limited strand dynamics. Surprisingly, while the strands of Caco-2 were the most dynamic, its strand resistance was comparable to that of the MDCK C7. In addition, the large difference in measured TER between MDCK II and C7 was also observed in the obtained strand resistances, which is in line with the current understanding that the TER is mainly defined by the conductive properties of the claudins, especially claudin-2, found in the strands [33, 74, 88]. The strand resistances of every epithelia were significantly higher than the strand break resistance. However, due to the rarity of the breaks, especially full openings, the strand resistances largely defined the overall TER, as shown by the sensitivity analysis. The MDCK C7 TER measured by Van Itallie et al. [33] was considerably smaller than the originally measured values (460 vs. 5650 Ω cm^2^) [84]. Our simulations show that this difference is explained by the cell size, since the C7 cells of Van Itallie et al. [33] are distinctly smaller than those of Gekle et al. [84], which leads to smaller length of bTJs and number of tTJ per area and thus higher TER. Further, although the claudin-2 dynamics model by Weber et al. [66] used a 3-strand model instead of the 4-strand used here and their description of the leak pathway differed from ours, there are surprising similarities. The calculated steady-state strand resistance defined for their claudin-2-transfected high-resistance MDCK I model equals to approximately 0.32 GΩ *μ*m, which is close to our claudin-2-containing MDCK II values.

Our models showed what kind of changes in the dynamic structure could lead to the observed experimental changes in the TJ barrier properties. Van Itallie et al. [34] found that ZO-1 stabilizes the TJ barrier, and therefore we described the decrease in stability caused by the ZO-1 knockdown by an increase in the strand break forming probability. The permeability results of the double ZO-1/2 knockdown study by Fanning et al. [54] were similarly replicated by changing this parameter. The higher change in the break forming probability in the double knockdown is in line with the observations that the two ZOs have redundant roles [54, 89], and thus knocking out both of them should decrease the strand stability even more. The decreased stability caused by the lack of binding between claudin-2 and ZO-1 was recently visualized in fibroblasts by Van Itallie et al. [50]. Interestingly, they found no difference in the number of breaks with or without this binding. However, this result might have been caused by the nonepithelial model system. On the other hand, we did not consider possible changes in the bTJ strand number or morphology due to the knockdowns. Umeda et al. [89] showed that ZO-1 knockout/ZO-2 knockdown completely eliminated the bTJ strands, accompanied by extremely low TER compared with the control. However, we expected that the strand number was not greatly affected by the ZO-1 and double ZO-1/2 knockdowns, since only a minor change in TER was reported [34, 54]. In addition, since we also had to increase the strand resistances to fit the TER model to these data, our results indicate that these knockdowns might also affect the strand resistance in a presently unknown manner.

The understanding regarding the role of the bTJ strand number in the properties of the epithelial barrier has changed over time. TER was originally found to depend exponentially on bTJ strand number [21]. This was hypothesized to arise from transient pores in the compartmentalized bTJ strand network [21, 90]. However, this idea has been refuted since TER is now known to mainly depend on the TJ claudin composition [5, 74, 88]. Our results agree that the dependence of TER on the strand number is not straightforward and show a complex dependence on strand resistance that is defined by the protein composition, the strand number, as well as the level of structural dynamics. The saturation towards higher strand numbers comes from the increasing relative role of the static tTJ pathway. Moreover, the results of TER as a function of time show the immensely varying behavior of the resistance in the epithelia with different levels of strand dynamics and numbers.

To the best of our knowledge, there have been no experimental studies that directly investigate the effect of strand number on molecular permeability. Colegio et al. [75] showed that an increase in strand number caused by the increased claudin-2 and -4 expression had no effect on the permeability of mannitol that diffuses via the leak pathway. However, based on their freeze-fracture images [75], the strand numbers were in the range of the permeability saturation shown in our simulations, and thus mannitol permeability should remain unchanged, indicating agreement with our results. The saturation in our results was caused by the tTJ pores, as the permeability of the bTJ pathway become lower than that of the tTJ pathway. The unaveraged permeability simulation raw data revealed vastly different permeability behavior depending on the strand numbers and levels of breaking dynamics. The observed biphasic behavior in the permeability simulations is most likely a property of the dynamic network system. In the high-permeability side of this behavior, the change in the number of the full opening events in the strand network caused by alterations in the strand number or level of dynamics led to large changes in permeability. However, since the low-permeability end of this behavior depends on the step-by-step diffusion between the compartments, the alterations in the strand number or dynamics have a lesser effect on the permeability.

Overall, our results concerning the effect of strand number, the ZO knockdowns, as well as the sensitivity analysis showed that changes in the bTJ structure or dynamics lead to considerably larger changes in the molecular permeability compared with the TER. Thus, measuring only the TER might hide important unseen changes in molecular permeability and in the barrier properties. The TER is a good indicator of epithelial condition and is straightforward to measure, but it contains a lot of uncertainty due to the differences in measurement setup and conditions. Our results strengthen the idea that both the molecular permeability and the TER are needed to properly define the TJ as a barrier [91, 92].

Our comparison with the steady-state models showed that they are not able to reproduce the same behavior as our dynamic models. Since there were constant breaks in the steady-state model, the changes in the strand number or the number of breaks had only a minor effect on the permeability and the TER. Nevertheless, our models had their limitations concerning both the chosen parameter values and the geometry. Because of the lack of rigorous experimental data, we were forced to estimate and fit the probabilities of the strand dynamics and build the model partially with assumptions and hypotheses available in the literature. Moreover, the TJ strand morphology is very diverse and heterogeneous, the number of strands varies within an epithelium [39, 55, 77, 78, 93], and the strands typically become tighter towards the apical direction [13]. Also, the expression of tricellulin differs between the epithelia, but the effects of its expression level on the TER and the permeability of molecules with similar size to 547-Da PEG were minor [16]. Further, many molecules permeate the epithelia through the cells using active transport processes, which are difficult to model due to their specificity. However, the PEG oligomers are hydrophilic [40], indicating that they mainly diffuse via the TJs. Thus, our model reflects the PEG-based permeability measurements. In addition, our model lacks the claudin-2 pore dynamics that have previously been observed [56, 66]. However, we opted to exclude these dynamics, since their effect has not been characterized for noncharged molecules. Finally, although there is evidence that the TJs form the main conduction pathway [27, 28], Günzel et al. [26] calculated that for many epithelia the transcellular resistance affects the overall TER or is even lower than the paracellular resistance. This indicates that our assumption that TJs define the TER will not work for every epithelium and condition.

We demonstrated that our dynamic structure models can reproduce the basic TJ barrier behavior. With the simplified structure and dynamics, the models enable the comparison between the molecular permeability and the TER under the same dynamic context at the level of the TJs. We showed that the TJ strand breaking dynamics can drastically alter both of these barrier properties independently of each other, highlighting the importance of both measures for characterizing the epithelial barrier. Furthermore, our results indicated that the leak pathway may be formed both by the tTJ pores and the bTJ strand dynamics with varying degrees, but differently for the permeability and the TER. Our models create a good methodological framework that can be used to integrate knowledge on TJ structure, parametrize experimental measurements, and produce hypotheses that can be studied experimentally. Refined versions of the models could include a more realistic strand network as well as inhomogeneities in the strands. This would provide tools to study how diseases that affect the TJ structure alter the properties of the epithelial barrier.

## Supporting information

S1 File Tight junctions permeability model. A Matlab model file of the tight junctions molecular permeability model.
(ZIP)

S2 File Tight junctions TER model. A Matlab model file of the tight junctions transepithelial electric resistance model.
(ZIP)
